# Serologic evidence of human monocytic and granulocytic ehrlichiosis in Israel.

**DOI:** 10.3201/eid0506.990605

**Published:** 1999

**Authors:** A Keysary, L Amram, G Keren, Z Sthoeger, I Potasman, A Jacob, C Strenger, J E Dawson, T Waner

**Affiliations:** Israel Institute for Biological Research, Ness Ziona, Israel.

## Abstract

We conducted a retrospective serosurvey of 1,000 persons in Israel who had fever of undetermined cause to look for Ehrlichia chaffeensis antibodies. Four of five cases with antibodies reactive to E. chaffeensis were diagnosed in the summer, when ticks are more active. All patients had influenzalike symptoms with high fever. None of the cases was fatal. Three serum samples were also seroreactive for antibodies to E. canis, and one was also reactive to the human granulocytic ehrlichiosis (HGE) agent. The titer to the HGE agent in this patient was higher than the serum titer to E. chaffeensis, and the Western blot analysis also indicated that the HGE agent was the primary cause of infection. We present the first serologic evidence that the agents of human monocytic ehrlichiosis (HME) and HGE are present in Israel. Therefore, human ehrlichiosis should be included in the differential diagnoses for persons in Israel who have been exposed to ticks and have influenzalike symptoms.


### Serologic Evidence of Human Monocytic and Granulocytic Ehrlichiosis in Israel

Avi Keysary,* Lili Amram,† Gershon Keren,‡ Zev Sthoeger,§ Israel Potasman,¶ Amir Jacob,# Carmella Strenger,* Jacqueline E. Dawson,** and Trevor Waner*

*Israel Institute for Biological Research, Ness Ziona, Israel; †Asaf Harofe Medical Center, Tzrifin, Israel; ‡Sheba Medical Center, Tel Hashomer, Israel; §Kaplan Hospital, Rehovot, Israel; ¶Bnei-Zion Medical Center, Haifa, Israel; #Schneider Children's Medical Center of Israel, Petach Tikvah, Israel; and **Centers for Disease Control and Prevention, Atlanta, Georgia, USA

We conducted a retrospective serosurvey of 1,000 persons in Israel who had fever of undetermined cause to look for *Ehrlichia chaffeensis* antibodies. Four of five cases with antibodies reactive to *E. chaffeensis* were diagnosed in the summer, when ticks are more active. All patients had influenzalike symptoms with high fever. None of the cases was fatal. Three serum samples were also seroreactive for antibodies to *E. canis,* and one was also reactive to the human granulocytic ehrlichiosis (HGE) agent. The titer to the HGE agent in this patient was higher than the serum titer to *E. chaffeensis,* and the Western blot analysis also indicated that the HGE agent was the primary cause of infection. We present the first serologic evidence that the agents of human monocytic ehrlichiosis (HME) and HGE are present in Israel. Therefore, human ehrlichiosis should be included in the differential diagnoses for persons in Israel who have been exposed to ticks and have influenzalike symptoms.

Human ehrlichiosis (HME) and human granulocytic ehrlichiosis (HGE), two emerging infectious diseases transmitted by ticks, are caused by *Ehrlichia chaffeensis* and the HGE agent of the *E. phagocytophila* genogroup, respectively. In the United States, HME and HGE were first described in 1987 and 1994, respectively (1). Since then, seroepidemologic studies have shown that these infections are also present in other parts of the world.

The first cases of HME and HGE were reported in Europe in 1991 and 1995, respectively (2,3). Serologic evidence of HGE has been found in Norway and Sweden (4). In South America, a case of *E. canis* infection was reported in Venezuela (5). One clinical case of HME has been reported in Mali, Africa (6). A serosurvey for HME of 756 patients from eight African countries suggested that the disease is rare in Africa (7).

Address for correspondence: Trevor Waner, Israel Institute for Biological Research, P.O. Box 19, Ness Ziona 70400, Israel; fax: 972-8-940-1443; e-mail: wanertnt@shani.net.

We describe the first serologic survey in Israel for HME and HGE, which documents the detection of antibodies reactive with HME and HGE agents.

### Materials and Methods

### Sera

One thousand serum samples from patients in Israel with fever of undetermined cause from 1994 to 1997 were received by the Israel National Reference Laboratory for Rickettsial Diseases. All specimens were serologically negative for Mediterranean spotted fever, murine typhus, and Q fever.

### Serology

The sera were tested retrospectively for immunoglobulin (Ig) G antibodies to *E. chaffeensis* and *E. canis* by indirect immunofluorescence antibody (IFA) (8). Briefly, DH82 cells heavily infected with the Israeli strain of *E. canis* (#611) (8) or the Arkansas strain of *E. chaffeensis* were pelleted and resuspended in growth medium.

Five microliters of the suspension were placed in each well of eight-well teflon-coated slides. The slides were dried at room temperature for approximately 30 minutes, fixed in acetone for 15 minutes, and then stored at 40°C. Serum samples were assayed for IgG by preparing and testing serum dilutions in PBS at their cutoff points of 1:64 for *E. chaffeensis* and 1:40 for *E. canis*. Positive sera were subsequently assayed at twofold dilutions. Positive control sera were provided by the Centers for Disease Control and Prevention (CDC).

Serum samples were sent to CDC for confirmation of results of the HME titers and for testing for HGE.

### Western Blot Analysis

One patient (#3) sample found positive for HGE was tested by Western blot at Johns Hopkins University, Baltimore, Maryland.

### Results

Of the 1,000 sera tested, five were found seropositive with HME (Table). During validation of the sera for HME antibodies, the CDC laboratory also found that one patient (#3) had an antibody titer of 1:2,048 to HGE. This sample was confirmed positive for HGE by Western blot.

None of the patients documented in this study had traveled overseas before their illness. All five cases occurred in persons, three male and two female, who lived on the coastal plain. Two of the three men lived in agricultural settlements. The average age of patients was 34.2 years (8 to 77 years). Four of the five patients were ill during summer.

The disease lasted up to 14 days. None of the cases was fatal. None of the patients reported being bitten by a tick. All patients had fever from 38.5°C to 40.2°C. Clinical signs were inconsistent: macular rash was present in only three patients and lymphomegaly in two. Four patients were leukopenic, and two were also thrombocytopenic. No changes in liver enzymes were detected in any of the patients.

The antibody titers to *E. chaffeensis* were 1:128 to 1:1,024. Similar results were obtained by CDC. Three of the five sera were also seropositive for *E. canis* antibodies; however, their titers were lower than those to *E. chaffeensis*.

Table. Clinical and serologic data for patients in Israel with antibodies to *Ehrlichia chaffeensis* and the human granulocytic ehrlichiosis agent

| | Patients | | | | |
|---|---|---|---|---|---|
| Clinical and serologic data | 1 | 2 | 3 | 4 | 5 |
| Date | 7/94 | 7/94 | 8/95 | 2/97 | 7/97 |
| Sex | F | F | M | M | M |
| Age (years) | 77 | 8 | 22 | 12 | 52 |
| IFA titer (HME) | 1:128 | 1:256 | 1:1,024 | 1:256 | 1:256 |
| IFA titer *E. canis* | 1:80 | 1:80 | 1:640 | <1:40 | <1:40 |
| IFA titer (HGE) | <1:64 | <1:64 | 1:2,048[a] | <1:64 | <1:64 |
| Body temperature (°C) | 40 | 39.2 | 40.2 | 40 | 38.5 |
| Symptoms: | | | | | |
| Vomiting | - | + | - | - | |
| Headache | | + | | ++ | |
| Chills | + | | + | | |
| Macular rash | + | | | + | + |
| Lymphomegaly | | + | + | | |
| Neck pain | - | + | - | - | - |
| Duration (days) | 7 | >5 | 12 | 14 | 14 |
| Tetracycline therapy | + | + | | + | - |
| Total leukocyte count/µl[b] | 3,700 | 4,000 | 3,500 | 4,000 | 16,700 |
| Total platelet count/µl[c] | 86,000 | 272,000 | 95,000 | 177,000 | 318,000 |

[a]Western blot analysis of the serum proved positive for HGE.
[b]Normal range for total leukocyte count 4,000-10,000/µl.
HGE, human granulocytic ehrlichiosis; HME, human monocytic ehrlichiosis; IFA, immunofluorescence assay.
[c]Normal range for total platelet count 150,000-450,000/µl.

### Conclusions

We retrospectively looked for *E. chaffeensis* antibodies in human patients with fever of undetermined cause. Four cases of HME were found, as well as a possible case of HGE with cross-reacting antibodies to HME.

Four of the five cases were diagnosed in summer, during peak tick activity. Patients' ages were 8 to 77 years. Symptoms were nonspecific, as has been described (1). All patients had influenzalike symptoms with high fever. Leukopenia was seen in four patients and thrombocytopenia in two; both these hematologic changes are typical of HME.

In our study three sera positive for *E. chaffeensis* were also seropositive for *E. canis*, unlike the African study in which all *E. chaffeensis*-positive sera were seronegative to *E. canis* in spite of the known strong cross-reactivity between the strains (7). The reason for this lack of cross-reactivity is unknown; however, reactivity in *E. chaffeensis* patients to *E. canis* antigens may develop only after prolonged exposure to the *Ehrlichia*, allowing expression of common antigens to be revealed.

In one seropositive *E. chaffeensis* case (#3), the titer to the HGE agent was higher than to *E. chaffeensis* and Western blot analysis for HGE was positive, which indicates that the HGE agent was the primary cause of infection. Serologic reactions with *E. chaffeensis* have been demonstrated after HGE infection (9). Cross-reaction between the two species of *Erhlichia* has been found in a small proportion of all HGE patients tested, which suggests that the causative *Ehrlichiae* share antigenic determinants.

Several tick species of the genus *Ixodes* are found in Israel, including the *I. ricinus*, which is the vector of the disease in Europe, and *I. redikorzevi,* which often bites humans (10,11). *Rhipicephalus sanguineus,* which is abundant in Israel, is a potential vector of HME agent. Coinfection by a number of tickborne diseases is not uncommon, and persons may be infected by both HME and HGE agents simultaneously.

A recent serosurvey of jackals in Israel tested against *E. canis, E. chaffeensis,* and *E. phagocytophila* genogroup antigens has shown that some jackals were seroreactive only to the *E. phagocytophila* genogroup antigen (12). The latter group of *Ehrlichia* consists of *E. equi, E. phagocytophila,* and the HGE agent (13). A close serologic and genetic relationship has been shown to exist among these three members, suggesting that they may be strains of a single species (1). The finding in jackals adds further evidence of one or more of the *E. phagocytophila* genogroup of *Ehrlichiae* in Israel.

In conclusion, we have presented the first serologic evidence that the agents of HME and HGE are present in Israel. Human ehrlichiosis should therefore be included in the differential diagnoses for persons in Israel who have been exposed to ticks and have influenzalike symptoms.

### Acknowledgments

We thank J.E. Dawson for her help in confirming the titers, and J.S. Dumler for the Western blot analysis.

Dr. Keysary is head of the Israel National Reference Laboratory of Rickettsial Diseases. His interests include diagnosis of infections caused by *Rickettsia, Coxiella,* and *Ehrlichia*.

### References

1. Walker DH, Babour AG, Oliver JH, Lane RS, Dumler JS, Dennis DT, et al. Emerging bacterial zoonotic and vector-borne diseases. JAMA 1996;275:463-9.
2. Morais JD, Dawson JE, Greene C, Filipe AR, Galhardas LC, Bacellar F. First European case of ehrlichiosis [letter]. Lancet 1991;338:633-4.
3. Brouqui P, Dumler JS, Leinhard R, Brossard M, Raoult D. Human granulocytic ehrlichiosis in Europe. Lancet 1995;346:782-3.
4. Bakken JS, Krueth J, Tilden RL, Dumler JS, Kristiansen BE. Serological evidence of human granulocytic ehrlichiosis in Norway. Eur J Clin Microbiol Infect Dis 1996;15:829-32.
5. Arraga-Alvarado C, Montero-Ojeda M, Bernardoni A, Parra 0. Human ehrlichiosis: report of the first case in Venezuela. Invest Clin 1996;37:35-49.
6. Uhaa IJ, MacLean JD, Greene CR, Fishbein DB. A case of human ehrlichiosis acquired in Mali: clinical and laboratory findings. Am J Trop Med Hyg 1992;46:161-4.
7. Brouqui P, Lecam C, Kelly PJ, Laurens R, Tounkara A, Sawadogo S, et al. Serologic evidence for human ehrlichiosis in Africa. Eur J Epidemiol 1994;10:695-8.
8. Keysary A, Waner T, Rozner M, Dawson JE, Zass R, Warner CK, et al. The first isolation, in vitro propagation, and genetic characterization of *Ehrlichia canis* in Israel. Vet Parasitol 1996;62:331-40.
9. Dumler JS, Brouqui P. Human granulocytic ehrlichiosis. In: Anderson B, Friedman H, Bendinelli M, editors. Rickettsial infection and immunity. New York and London: Plenum Press; 1997. p. 149-61.
10. Reed KD, Mitchell PD, Persing DH, Cameron V. Transmission of human granulocytic ehrlichiosis. JAMA 1995;273:23.
11. Theodor O, Costa M. In: Ectoparasites. A survey of the parasites of wild mammals and birds in Israel. Part 1. Jerusalem: The Israel Academy of Sciences and Humanities; 1997. p. 92-103.

12. Waner T, Beneth G, Strenger C, Keysary A, King R, Harrus S. Antibodies reactive with *Ehrlichia canis, Ehrlichia phagocytophila* genogroup antigens and the spotted fever group antigens, in free-ranging jackals (*Canis aureus syriacus)* from Israel. Vet Parasitol 1999;82:121-8.
13. Dumler JS, Asanovich KM, Bakken JS, Richter P, Kimsey R, Madigan JE. Serologic cross-reactions among *Ehrlichia equi, Ehrlichia phagocytophila,* and human granulocytic ehrlichia. J Clin Microbiol 1995;33:1098-103.
